# Molecular characterization of beta-tubulin from *Phakopsora pachyrhizi*, the causal agent of Asian soybean rust

**DOI:** 10.1590/S1415-47572010005000040

**Published:** 2010-06-01

**Authors:** Talles Eduardo Ferreira Maciel, Maíra Cristina Menezes Freire, Álvaro M. R. de Almeida, Luiz Orlando de Oliveira

**Affiliations:** 1 Departamento de Bioquímica e Biologia Molecular, Universidade Federal de Viçosa, Viçosa, MGBrazil; 2 Empresa Brasileira de Pesquisa Agropecuária, Centro Nacional de Pesquisa de Soja, Londrina, PRBrazil

**Keywords:** Asian soybean rust, beta-tubulin, genetic diversity, *Phakopsora pachyrhizi*, soybeans

## Abstract

β-tubulins are structural components of microtubules and the targets of benzimidazole fungicides used to control many diseases of agricultural importance. Intron polymorphisms in the intron-rich genes of these proteins have been used in phylogeographic investigations of phytopathogenic fungi. In this work, we sequenced 2764 nucleotides of the β-tubulin gene (Pp tubB) in samples of *Phakopsora pachyrhizi* collected from seven soybean fields in Brazil. Pp tubB contained an open reading frame of 1341 nucleotides, including nine exons and eight introns. Exon length varied from 14 to 880 nucleotides, whereas intron length varied from 76 to 102 nucleotides. The presence of only four polymorphic sites limited the usefulness of Pp tubB for phylogeographic studies in *P. pachyrhizi*. The gene structures of Pp tubB and orthologous β-tubulin genes of *Melampsora lini* and *Uromyces viciae-fabae* were highly conserved. The amino acid substitutions in β-tubulin proteins associated with the onset of benzimidazole resistance in model organisms, especially at His _6_ , Glu _198_ and Phe _200_ , were absent from the predicted sequence of the *P. pachyrhizi* β-tubulin protein.

*Phakopsora pachyrhizi* Sydow & Sydow, the causal agent of Asian soybean rust, is an obligate, biotrophic plant pathogen initially identified in Japan (Henning, 1903) and nearly a 100 years later in Brazil (Yorinori and Paiva, 2002), where it accounts for 30%-75% of soybean losses in the field. Asian soybean rust has been managed with the use of strobilurin, triazole and benzimidazole fungicides (Soares *et al.*, 2004). However, alternatives are necessary because the use of fungicides increases production cost, is environment unfriendly, and may trigger pathogen resistance. The challenges are enormous as no geographic region or state is free of Asian soybean rust and no resistant variety has yet been released. In previous work, we reported evidence that supported an African origin for *P. pachyrhizi* found in Brazil and suggested that multiple, independent, long-distance dispersal events were a plausible mechanism of introduction (Freire *et al.*, 2008). For a species that was introduced recently and that appears to be maintained asexually (Yorinori and Paiva, 2002), the internal transcribed spacer regions (ITS) contain a surprisingly high level of intraspecific sequence variation (Freire *et al.*, 2008). To refine our inferences on the geographic patterns of genetic variation in Brazilian *P. pachyrhizi* complementary analyses with a less polymorphic region are necessary.

As the major components of microtubules, α-tubulins and β-tubulins have important cellular functions and are amongst the most highly conserved eukaryotic proteins (Wade, 2007). Moreover, β-tubulins are a target for benzimidazole fungicides (Davidse, 1986). The mechanism of resistance to benzimidazole compounds has been associated with specific amino acid changes in the β-tubulin of organisms such as *Aspergillus nidulans* and *Neurosporacrassa* (Jung *et al.*, 1992; Cooley and Caten, 1993; Oakley, 2004). Despite the high level of conservation at the protein level, the β-tubulin genes are intron-rich and are therefore potentially useful in fungal phylogeography (O'Donnell *et al.*, 2000; Ceresini *et al.*, 2007; Kauserud and Shalchian-Tabrizi, 2007; Konrad *et al.*, 2007).

In this report, we (1) describe and analyze the complete sequence of the β-tubulin gene of *P. pachyrhizi* (Pp tubB), (2) compare the gene structure of Pp tubB and the orthologous β-tubulin genes of the rust pathogens *Melampsora lini* and *Uromyces viciae-fabae*, (3) discuss the amino acid sequence alignments and phylogenetic analyses of β-tubulins of *P. pachyrhizi*, other rust pathogens, and model organisms, and (4) identify specific amino acid residues of β-tubulin potentially associated with benzimidazole resistance in *P. pachyrhizi*.

By using the gene sequence of the *M. lini* β-tubulin gene (GenBank: AF317682) (Ayliffe *et al.*, 2001) as the initial query, we ran a BLAST search (Altschul *et al.*, 1990) against public databases at the National Center for Biotechnology Information. The search retrieved several highly similar sequences (E-values = 0.0) of filamentous fungi, especially basidiomycetes. Among the sequences retrieved from GenBank was a 40,837 nucleotide genomic clone of *P. pachyrhizi*. This clone (JGIAFNA-573F20a; GenBank AC 170158) contained a segment with 84% identity to the query sequence. Further refinement of the sequence alignments showed that this segment contained sequence features which were highly conserved among β-tubulin genes of basidiomycetes (data not shown). This segment was fed into the program Primer3 (Rozen and Skaletsky, 2000), and specific primers were designed for PCR amplification of the entire coding sequence, intervenient sequences, and adjacent flanking regions at the 5' and 3' termini of the Pp tubB. With the assistance of the program PrimerSelect (DNASTAR, Madison, WI, EUA), ten primer sequences were selected (1: 5'-GACACGGTAAGGGCTTGAGT-3'; 2: 5'-CAAGGTGCTTCCCACATACC-3'; 3: 5'-CCTCC AAAGTGTCAGTCAAAC-3'; 4: 5'-CGGGGTACATAC TTGTTGGC-3'; 5: 5'-CTCGATCAGGTACAAGGGAA C-3'; 6: 5'-TCAAACATCTGGGAGGTCAG-3'; 7: 5'-CCCTACAATGCGGTTCTCTC-3'; 8: 5'-GTTGGACTC AGCCTCTGTGA-3'; 9: 5'-GTGGCAGCTTATTTCAG GGG-3'; 10: 5'-CCAATTCCCTVTGTTACTGA-3').

*Phakopsora*-infected soybean leaves were collected from seven soybean fields (Table 1). Uredinospores were harvested and total genomic DNA was extracted according to Freire *et al.* (2008). PCR amplifications of the Pp tubB were done with the primers indicated above, in five combinations (1-2, 3-4, 5-6, 7-8 and 9-10). Electrophoresis in agarose gels indicated that the reactions each yielded a single fragment of the expected size. Non-incorporated nucleotides and primers were removed using EXOSAP (USB Corporation, Cleveland, OH, USA)).

The primers used in the original PCR amplification were also used for sequencing. All of the sequences were imported into SEQUENCHER version 4.8 (Gene Codes Corp., Ann Arbor, MI, USA) for editing and DNA sequence alignment. A consensus sequence for the entire Pp tubB, including flaking regions, was generated for each sampling site. The relative positions of the initiation codon, exons, introns and termination codon in Pp tubB were inferred from alignments with published orthologous gene copies of other basidiomycetous fungi that were used as guides. DNA sequences were translated into protein sequences using the universal code after removing the introns. A dataset of protein sequences was constructed using the program T-COFFEE (Notredame *et al.*, 2000) with default values. The phylogenetic relationships of Pp tubB protein with β-tubulins of other filamentous fungi, *Saccharomycescerevisiae* and *Glycine max* were inferred using the neighbor-joining method as implemented in MEGA version 4.0 (Tamura *et al.*, 2007). Bootstrap values were obtained with 1000 replicates.

Our strategy for sequencing the Pp tubB yielded five overlapping DNA fragments that resulted in a total aligned sequence of 2764 nucleotides after assembly. The sequence was deposited in GenBank with accession number GU354165. An initiation codon (ATG) and a termination codon (TAG) delimited an open reading frame of 1341 nucleotides flanked by segments of 401 and 234 nucleotides 5' and 3' to the coding region, respectively. The promoter region contained the signals characteristically involved in the initiation of transcription, such as three putative TATA boxes located at -176, -320 and -386 and five putative CAAT boxes located at -92, -229, -237, -269 and -350. A pyrimidine-rich element of 30 nucleotides was found in the promoter region between -205 and -175. Such a pyrimidine-rich region is commonly associated with potential transcription factor binding sites in many genes of filamentous fungi (Cooley and Caten, 1993). The AATAAA recognition signal associated with the efficient formation of mRNA 3' termini (Wahle and Rüegsegger, 1999) was present 44 bases downstream from the termination codon.

The Pp tubB gene contained nine exons (14, 19, 16, 55, 49, 880, 172, 121 and 15 nucleotides long, respectively) and eight introns (125, 76, 86, 102, 85, 84, 99 and 128 nucleotides long, respectively). The consensus sequences GT(A/g)NGT at the 5' terminus and (C/T)AG at the 3' terminus flanked the introns. These consensus sequences are found at the intron-exon boundaries of many filamentous fungi (Begerow *et al.*, 2004). The seven Pp tubB sequences diverged at only four polymorphic sites. We found a 1 bp indel in a polyT at 1580, which was either nine or ten bases long. The presence of sequences differing in length resulted in peak displacements and chaotic electrophoretograms when this polyT was reached during the sequencing of some samples. We also found three base substitutions, identified as ambiguities: R (G or A) upstream from the initiation codon at position -50, M (A or C) in the second intron at 231, and Y (C or T) in the third intron at 323. These three polymorphic sites were identified as overlapping double peaks in electrophoretograms of both strands. These double peaks appeared in electrophoretograms of DNA sampled from distinct fields, which suggested that our samples contained a pool of strains and that distinct fields shared these strains. Although the Pp tubB gene contained a large number of introns and intron length varied substantially, the phylogeographic usefulness of these features in *P. pachyrhizi* was hampered by the extremely low levels of intron variability within and among the seven soybean fields sampled. For some organisms, such as *Coniophora arida* and *Coniophora olivacea* (Boletales), the ITS region is less polymorphic than in β-tubulin genes (Kauserud and Shalchian-Tabrizi, 2007). The lack of polymorphic sites in the intron-rich Pp tubB gene contrasted sharply with the high level of sequence variation we found in the ITS1 and ITS2 regions (Freire *et al.*, 2008).

The gene structures of Pp tubB and orthologous β-tubulin genes of *M. lini* and *U. viciae-fabae* were highly conserved in length, distribution, and intron and exon insertion sites (data not shown). Currently, *M. lini* and *U. viciae-fabae* are the only two rust pathogens for which complete β-tubulin gene sequences available in public databases. Although intron length and position were conserved, intron sequences were highly divergent among these three rust species. In most attempts, sequence alignments among introns of equivalent position simply were not achievable. For those that did align, the maximum identity was < 30%. The number of introns in other fungal β-tubulin genes varies considerably. For example, there are only three introns in the *Septoria nodorum* β-tubulin gene (Cooley and Caten, 1993) whereas the β-tubulin genes of many basidiomycetous fungi contain 6-10 introns (Ayliffe *et al.*, 2001).

The Pp tubB gene encoded a full-length protein of 447 amino acids (Figure 1). A search at the conserved domain database (Marchler-Bauer *et al.*, 2007) revealed that the *P. pachyrhizi* β-tubulin protein (Pp TUB) had four domains characteristically found in other β-tubulins: a nucleotide-binding site, taxol-binding site, an α/β domain interface and a β/α domain interface. Sequence alignment using the BLAST algorithm (Altschul *et al.*, 1990) revealed that Pp TUB shared high levels of sequence identity with β-tubulin from other basidiomycetous fungi: *U. viciae-fabae* (98%), *M. lini* (96%), and *Pleurotus sajor-caju* (89%), other Ascomycota: *N. crassa* (82%), *A. nidulans* (82%), and *S. cerevisiae* (73%), higher animal species, such as *Bos taurus* (85%) and higher plant species, such as *G. max* (40%). The amino acid sequence alignment of β-tubulins indicated that sequence heterogeneity was greatest in the carboxy-termini. This higher level of sequence divergence in the carboxy-termini has been associated with the location of this region at the surface of the heterodimers, its role as a binding surface for microtubule-associated proteins, and its function as a target for post-translational modifications (Wade, 2007).

The mechanism of resistance to benzimidazole compounds has been associated with specific amino acid substitutions in the β-tubulin of many organisms. The benzimidazole sensitivity of microsporidia such as *Vittaforma corneae* and *Enterocytozoon bieneusi* is related to the presence of six amino acids: His_6_, Ala_165_, Phe_167_, Glu_198_, Phe_200_ and Arg_241_ (Franzen and Salzberger, 2008). Resistance to benzimidazole in a *S. nodorum* mutant arose as a result of a base substitution at the first position of codon 6 (C → T) of the β-tubulin gene, which led to the amino acid substitution His_6_ → Tyr_6_ (Cooley and Caten, 1993). Likewise, benzimidazole resistance in a *N. crassa* mutant appeared as a result of an amino acid substitution at another site (Glu_198_ → Gly_198_) (Fujimura *et al.*, 1992). Most interestingly, experiments reported by Jung *et al.* (1992) with *A. nidulans* showed that amino acid substitutions at some sites, such as Ala_165_, conferred benzimidazole resistance but lead to abnormal cell growth, whereas amino acid substitutions at three other site (His_6_, Glu_198_ and Phe_200_) conferred resistance to benzimidazole agents without causing any conditional blockage of cell growth. Given that β-tubulins are amongst the most highly conserved eukaryotic proteins, Oakley (2004) fitted the amino acid sequences of *A. nidulans* α- and β-tubulins to the three-dimensional structure of *Bos taurus* α- and β-tubulins (Nogales *et al.*, 1998) and showed that amino acids associated with benzimidazole resistance in *A. nidulans* (His_6_, Tyr_50_, Gln_134_, Ala_165_, Glu_198_, Phe_200_ and Met_257_) were structurally close together; this finding suggested that the binding site for benzimidazole agents may be in the region defined by these mutations.

To the best of our knowledge, no studies have examined the relationship between amino acid substitutions and benzimidazole resistance in *P. pachyrhizi*. As shown here, the amino acid substitutions in β-tubulin associated with benzimidazole resistance in closely related organisms were absent in the predicted Pp TUB sequences from the *P. pachyrhizi* samples collected in seven Brazilian soybean fields. Nevertheless, we found a high level of sequence identity between Pp TUB and β-tubulins of model organisms such as *A. nidulans* and *N. crassa* (Figures 1  and 2), for which there is a clear relationship between amino acid substitutions at given sites and benzimidazole resistance (Jung *et al.*, 1992; Cooley and Caten, 1993; Oakley, 2004). We hypothesize that the emergence of resistance in *P. pachyrhizi* is likely to follow a similar pattern of amino acid substitutions in Pp TUB. However, we cannot exclude the possibility that there were no benzimidazole resistant mutants in the soybean fields at the time of sampling. Because of the limitations inherent to our sampling and sequencing strategies, we can only imply that if mutants were indeed present, their frequencies were extremely low. The appearance of natural mutants with amino acid substitutions in Pp TUB, especially at His_6_, Glu_198_ and Phe_200_, should be closely monitored since the continual use of benzimidazoles for controlling Asian soybean rust may well increase the selective pressure under field conditions in the near future.

**Figure 1:**
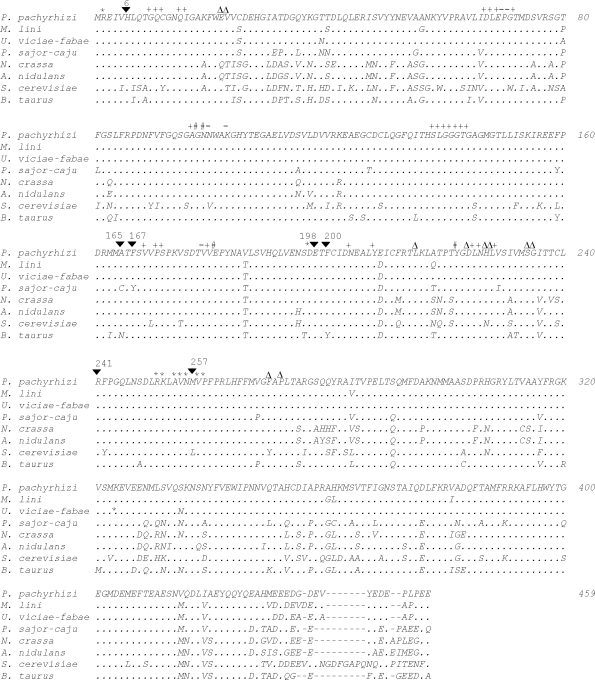
T-Coffee alignment of β-tubulin proteins. The amino acid residues are shown in single-letter code. Dots indicate residues identical to those of *Phakopsora pachyrhizi* β-tubulin and hyphens indicate gaps. Conserved domains are indicated as follows: (*) beta/alpha domain interface, (-) alpha/beta domain interface, (#) alpha/beta and beta/alpha domain interface, (+) nucleotide binding site and (Δ) taxol binding site. Black inverted triangles indicate amino acids thought to be associated with benzimidazole sensitivity in *A. nidulans*. GenBank accession numbers are: *P. pachyrhizi* (GU354165, this work), *M. lini* (Q9HFQ3), *U. viciae-fabae* (Q96TU8), *P. sajor-caju* (AAD21093), *N. crassa* (XP_957669), *A. nidulans* (XP_658786), *S. cerevisiae* (V01296) and *B. taurus* (1JFF).

**Figure 2:**
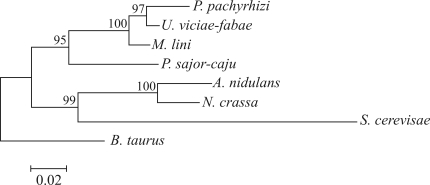
Neighbor-joining tree based on β-tubulin proteins. Relative branch lengths are shown to scale. Bootstrap values (1000 replicates) are indicated. GenBank accession numbers are: *P. pachyrhizi* (GU354165, this work), *M. lini* (Q9HFQ3), *U. viciae-fabae* (Q96TU8), *P. sajor-caju* (AAD21093), *N. crassa* (XP_957669), *A. nidulans* (XP_658786), *S. cerevisiae* (V01296) and *B. taurus* (1JFF).

## Figures and Tables

**Table 1:** Geographical location of the seven soybean fields from which *Phakopsora*-infected soybean leaves were collected.

Soybean field	State	Latitude	Longitude
Chapadão do Sul	Mato Grosso do Sul	-18°47'39''	52°37'22''
Guarda Mor	Minas Gerais	-17°46'15''	47°05'54''
Palmas	Tocantins	-10°12'46''	48°21'37''
Piracicaba	São Paulo	-22°43'31''	47°38'57''
Rio Verde	Goiás	-17°47'53''	50°55'41''
Seberi	Rio Grande do Sul	-27°28'41''	53°24'09''
Viçosa	Minas Gerais	-20°45'14''	42°52'55''
